# Failure of gamma knife radiosurgery for sporadic vestibular schwannomas: a systematic review and meta-analysis

**DOI:** 10.1007/s00405-025-09527-1

**Published:** 2025-06-24

**Authors:** April N. Taniguchi-Lo, Christian M. Shannon, Charlotte I. Rivers, Shaun A. Nguyen, Paul R. Lambert

**Affiliations:** 1 Department of Otolaryngology - Head and Neck Surgery, Medical University of South Carolina, 135 Rutledge Avenue, MSC550, Charleston, SC 29425 USA; 2 College of Medicine, University of Central Florida, Orlando, FL USA

**Keywords:** Vestibulocochlear, Gamma knife, Radiosurgery, Vestibular schwannoma, Failure

## Abstract

**Purpose:**

This review aims to characterize the limitations of Gamma Knife radiosurgery (GKRS) with investigation of failure rates for primary and salvage (post-surgical) cases, identification of risk factors associated with increased rates of treatment failure, and evaluation adverse outcomes.

**Methods:**

A systematic search (Cochrane Library, PubMed, SCOPUS, and CINAHL) of studies on sporadic vestibular schwannomas treated with Gamma Knife radiosurgery was conducted in accordance with PRISMA guidelines. Primary outcome measures included continuous measures (mean), proportions (%), and relative risks (RR) with 95% confidence intervals (CI).

**Results:**

Forty-three studies (*n* = 5619 patients) were included. The proportion of GKRS failure for primary (6.4% [95% CI 4.8–7.7]) and salvage treatment (9.0% [5.9–12.8]) did not differ (2.8%, [-0.2-6.9], *p* = 0.06). When stratified by various thresholds on imaging, an increase of greater than 15% in a tumor’s volume was seen 3.2% [2.1–4.6] which was less than rates in other volumetric thresholds of failure (*p* = 0.0001). Treatment edema identified by brainstem hyperintensity on imaging was the most frequent toxicity (8.8% [7.1–10.8]) with symptomatic management in about 1 in 4 cases. New onset cranial nerve adverse outcomes occurred for trigeminal neuralgia 4.9% [2.8–7.6%], trigeminal numbness 4.3% [2.7–6.2%], and temporary facial paresis 3.2% [1.3–5.9%].

**Conclusion:**

Gamma Knife radiosurgery is an effective treatment for sporadic vestibular schwannomas with low rates of failure. Our findings suggest failure rates are not affected by treatment timing and comparable for the smaller sized tumors. Gamma Knife radiosurgery is safe with minimal adverse outcomes such as cranial nerve dysfunction, required retreatment, or other radiation toxicity.

**Supplementary Information:**

The online version contains supplementary material available at 10.1007/s00405-025-09527-1.

## Introduction

Vestibular schwannomas (VS) are benign vestibulocochlear nerve sheath tumors with an increasing incidence of 3–5 cases per 100,000 person-years [1, 2]. These tumors can be asymptomatic or present as asymmetric hearing loss, unilateral tinnitus, ataxia, vertigo, or headache [3]. With a variable clinical course, VS management ranges from observation, stereotactic radiosurgery (SRS), microsurgery, or a combination of these strategies [4].

SRS is a safe, effective treatment with comparable long-term quality of life outcomes and tumor control rates for smaller tumors [5–7]. The high dose radiotherapy is often administered via gamma rays as Gamma Knife radiosurgery (GKRS) or megavoltage x-rays as part of a linear accelerator (LINAC) system with comparable outcomes [8]. GKRS is the most well-known modality to achieve VS tumor control, yet its failure rate ranges from 2.4 to 14.7% [9–11]. It is difficult to assess GKRS outcomes because tumor control is frequently defined differently amongst studies.

This study aims to quantify the rate of failure for GKRS in sporadic VS and investigate potential factors associated with higher rates of treatment failure. A secondary goal was to assess treatment outcomes including toxicities associated with GKRS.

## Methods

This research was conducted in accordance with the principles outlined in the Declaration of Helsinki. Institutional Review Board approval was not required since this study reviewed published literature. Informed consent was not applicable.

### Search criteria

**Fig. 1:**
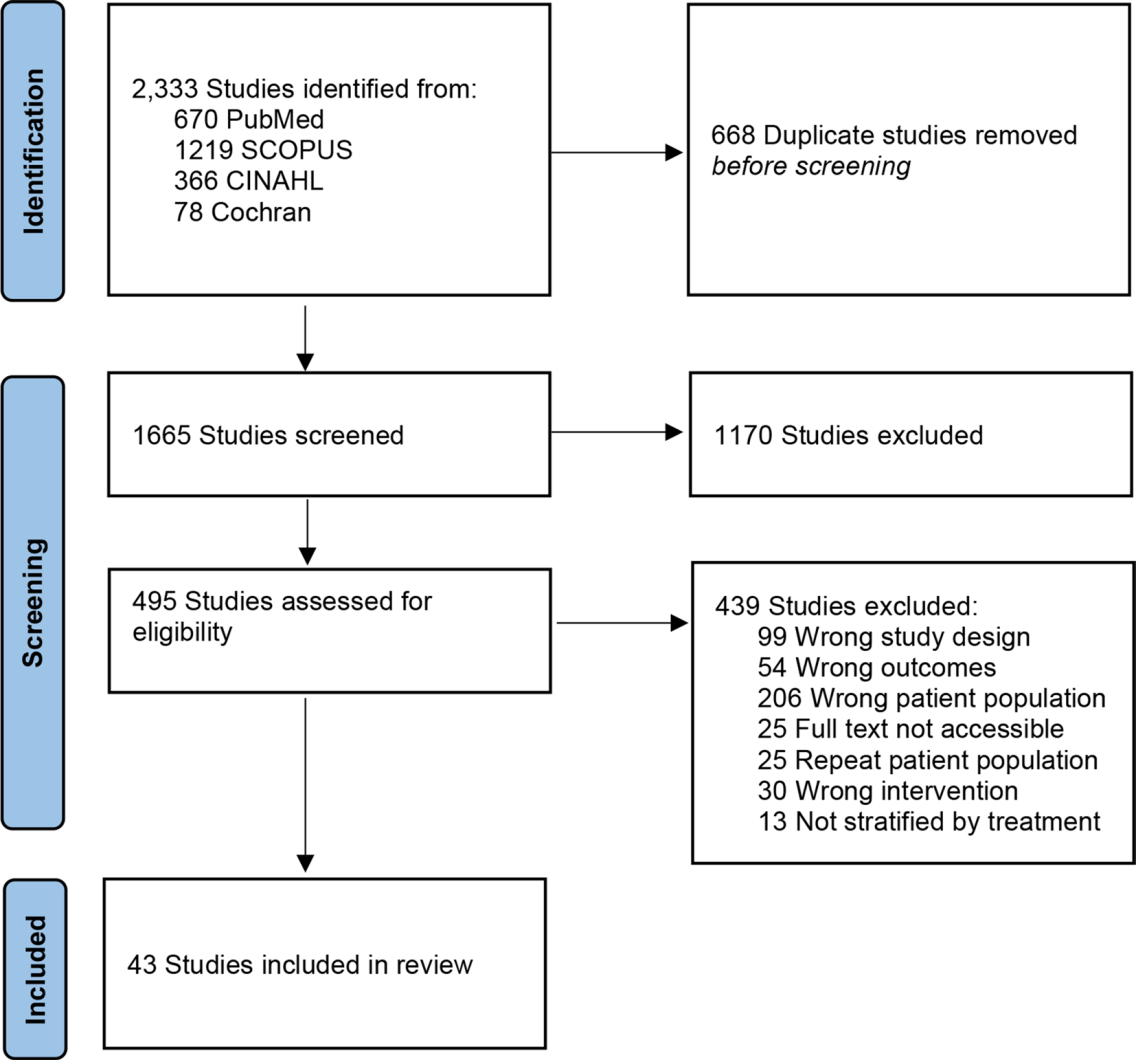
Preferred reporting items for systematic reviews and meta-analysis (PRISMA) flow diagram outlining the study screening process

This study was conducted according to the Preferred Reporting Items for Systematic Reviews and Meta-analyses: the PRISMA guidelines [12]. To identify studies for inclusion, two authors (A.N.T. and C.M.S.) created a detailed search strategy for the following databases: PubMed (US National Library of Medicine, National Institutes of Health), SCOPUS (Elsevier), Cumulated Index in Nursing and Allied Health Literature (CINAHL - EBSCO), and Cochrane Library (Wiley). The search strategies used a combination of subject headings (e.g. Medical Subject Headings [MeSH] in PubMed) and keywords for the following concepts: Gamma Knife, Stereotactic Radiation, and Acoustic Neuroma or Vestibular Schwannoma. The PubMed search strategy was modified for the other databases by replacing MeSH terms with appropriate subject headings, if available, and maintaining similar keywords. To identify additional articles, the reference lists of relevant articles were hand-searched. References were exported into Covidence (Veritas Health Innovation Ltd), the review management software, for study selection as shown in (Fig. 1).

### Selection criteria

Studies of pediatric and adult patients with VS treated by single fraction GKRS were included. Patients were excluded if they had Neurofibromatosis 2 (NF2) or a comorbid brain tumor (e.g. facial schwannoma, meningioma) to minimize confounding effects of concurrent intracranial treatments. Excluded studies were non-English, case reports, reviews, machine-learning studies, duplicates, inaccessible articles, or those with missing statistical data. Studies were ineligible if the tumor failure rate was not identified or if patients had received a prior course of SRS. Two reviewers (A.N.T. and C.M.S.) independently assessed abstracts to identify all articles that met the inclusion criteria and then applied exclusion criteria to full text review. Conflicts were resolved by a third reviewer (S.A.N.).

### Data extraction

Two reviewers (A.N.T. and C.M.S.) independently conducted data extraction. Data extracted included author, publication year, study design, patient demographics (age, gender, tumor characteristics), Koos classification, stereotactic treatment specifications, treatment failure rates, and toxicity rates. Disagreements were resolved by a third reviewer (S.A.N.).

The Koos classification stratifies tumors according to location and effect on local structures. Grade I tumors are in the internal auditory canal (IAC), Grade II tumors have extrameatal extension, Grade III tumors are intra- and extrameatal that contact the brainstem, and Grade IV tumors result in brainstem compression [13]. Based on a prior study, tumor volume at treatment initiation was organized into the following groups: < 0.8 cm^3^, 0.8-< 1.6 cm^3^, 1.6-< 3.2 cm^3^, and ≥ 3.2 cm^3^ [14]. Mean tumor volume was used to sort into the outlined categories. Serviceable hearing was defined by Gardner-Robertson (GR) grade I/II or the American Academy of Otolaryngology-Head and Neck Surgery (AAO-HNS) hearing class A/B [15].

Treatment failure was defined by radiologic evidence of persistent tumor growth that was differentiated from transient post-radiation edema. The most reported thresholds for failure were growth greater than 2 mm in a single plane, greater than a 10% volume increase, or greater than a 20% volume increase. However, 18 studies did not report a specific definition of tumor growth. Salvage treatment was defined as the use of SRS after an initial failed treatment of microsurgery. Magnetic Resonance Imaging (MRI) T2-weighted hyperintensity in the brainstem was interpreted to represent treatment related edema.

Articles that met criteria were critically appraised to assess level of evidence using the Oxford Center for Evidence-Based Medicine criteria [16]. The risk of bias was assessed according to Risk of Bias in Non-Randomized Studies—of Interventions (ROBINS-I) tool to evaluate non-randomized studies [17]. Two authors (A.N.T. and C.M.S.) performed independent risk assessments for the included studies. Risk of bias items assessed were: bias due to confounding, bias in selection of participants into the study, bias in classification of interventions, bias due to deviations from intended interventions, bias due to missing data, bias in measurement of outcomes, and bias in selection of the reported results. The risk of bias for each aspect was graded as low, unclear, or high.

### Statistical analysis

Meta-analysis of continuous measures (age) was performed by Comprehensive Meta-Analysis version 4 (Biostat Inc., Englewood, NJ, USA). Meta-analysis of proportions (failure rates) and relative risk (GKRS treatment failure vs. microsurgery) was performed using MedCalc 20.110 (MedCalc Software Ltd., Ostend, Belgium). Each measure (mean/proportion (%)/relative risk (RR) and 95% confidence interval (CI)) was weighted according to the number of patients affected. Heterogeneity among studies was assessed using I^2^ statistics with fixed-effects (I^2^ < 50%) and random-effects (I^2^ > 50%). In addition, a comparison of weighted proportions was done to compare failure rates. Potential publication bias was evaluated by visual inspection of the funnel plot, as bias results in asymmetry of the funnel plot, and Egger’s test, which statistically examines this asymmetry [18]– [19]. A p-value of < 0.05 was considered to indicate a statistically significant difference for all statistical tests.

## Results

### Search and study characteristics

**Fig. 2:**
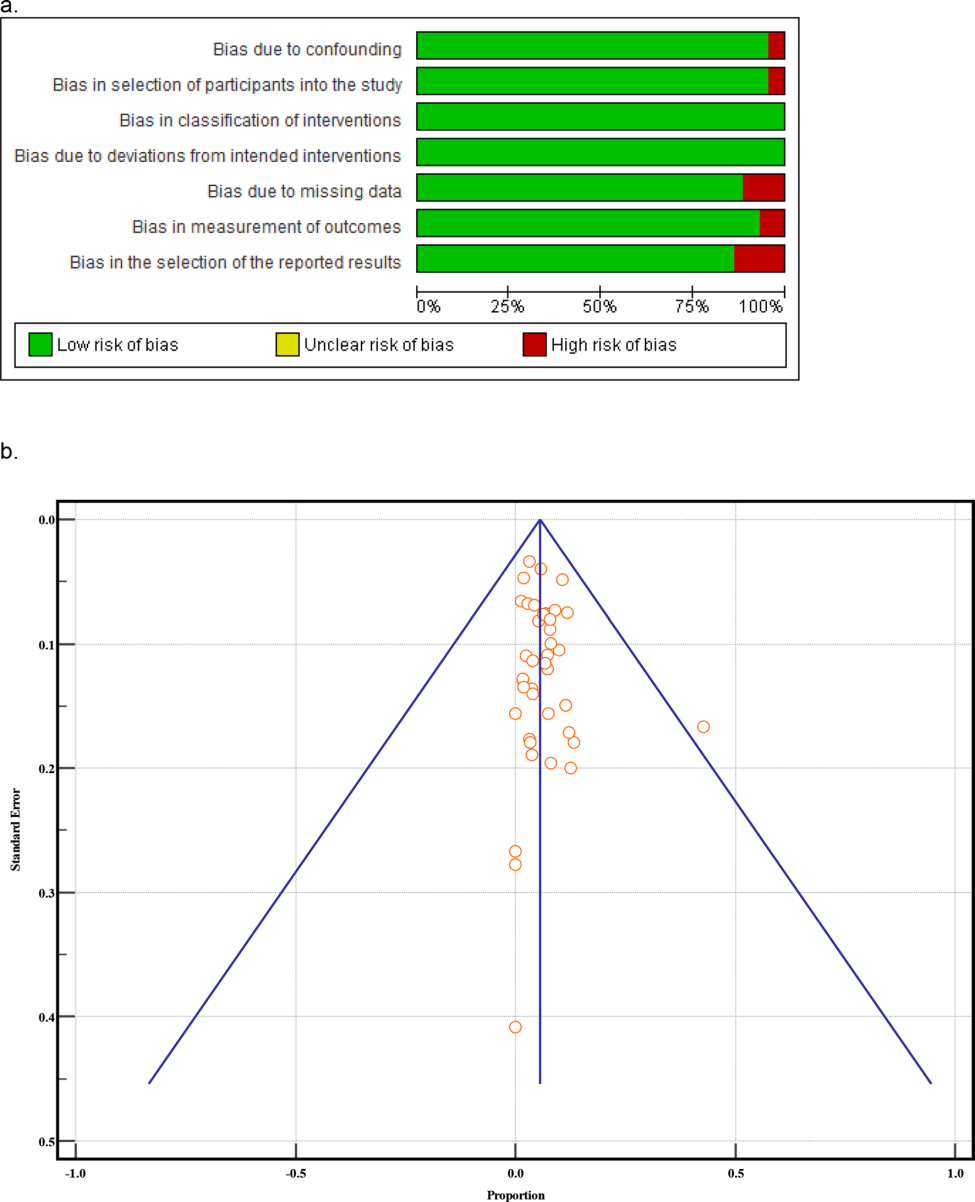
Risk of bias **a** Risk of bias evaluation by authors. Each potential arena for bias is portrayed as percentages among the included studies **b** Funnel plot of the meta-analysis to investigate publication bias

As seen in Fig. 1, the literature search identified 1665 unique studies. Title and abstract screening excluded 1170 studies with 495 studies reviewed for full-text eligibility. A total of 43 studies (*N* = 5619) published from 2002 to 2023 met inclusion criteria and were mainly retrospective cohort studies, Oxford Level of Evidence 4 (Table 1). Most studies were evaluated as low risk, with greater potential for bias due to missing data and bias in selection of the reported results (Fig. 2). A funnel plot with Egger’s test (1.0, [95% CI − 0.2–2.2], *p* = 0.09) demonstrated all studies were within the funnel except for one with little asymmetry, suggesting little publication bias.

**Table 1:** Characteristics of included studies

Study, Year	OLE^a^	Study Design	Tumors (*n*)	Treatment failure (*n*)	Failure definition^b^	Median Dose (range) (Gy)
Bailo 2016	4	Retrospective	59	1	> 10% vol^c^ or ≥ 2 mm growth	13 (11–15)
Bailo 2017	4	Retrospective	90	9	> 10% vol or ≥ 2 mm growth	13 (11.6–14)
Baschnagel 2013	4	Prospective	40	0	> 2 mm growth or retreatment	12.5 (12.5–13)
Brown 2011	4	Retrospective	53	2	growth	12.5 (12.5–13)
Carlson 2013	4	Retrospective	44	5	growth	(12–13)
Coelho 2008	4	Retrospective	12	0	growth	
Dumot 2022	4	Retrospective	175	12	> 20% vol	12
Dumot 2023	4	Retrospective	150	8	> 20% vol	12
Fayad 2009	3	Prospective	60	1	1.7 fold vol	12
Franzin 2009	4	Retrospective	50	2	growth	13 (12–16)
Golfinos 2016	3	Retrospective	83	6	growth	(12–13)
Han 2012	4	Retrospective	444	8	growth	13 (12–16)
Haque 2011	4	Retrospective	232	3	> 5 mm growth	12 (8–20)
Huang 2017	4	Retrospective	173	14	> 2 mm growth	13 (11–20)
Johnson 2019	4	Retrospective	871	27	> 15% vol	13 (8–20)
Killeen 2022	4	Retrospective	35	15	> 20% vol or 2 mm growth	13
Kim 2015	3	Cross-sectional	27	1	growth	13.1 (11–14)
Klijn 2016	4	Retrospective	420	45	growth or visual impairment	11 (11–13)
Massager 2006	4	Retrospective	82	2	growth	12
Mousavi 2015	4	Retrospective	68	5	growth	12.5
Muacevic 2004	4	Retrospective	219	6	growth	13 (10–15)
Ogino 2021 (I)	4	Retrospective	209	9	growth	12.5 (11–25)
Ogino 2021 (IV)	4	Retrospective	170	11	growth	12.5 (10.5–22)
Ottaviani 2002	4	Prospective	30	4	growth	13.4 (12–14)
Ozer 2022	4	Prospective	24	3	continuous enlargement	12.5
Paek 2005	4	Prospective	25	2	growth	12 (11–14)
Park 2011	4	Case control	31	1	> 15% vol	14.2 (1.2)
Peker 2023	4	Retrospective	54	1	> 20% vol	12 (10–12)
Pikis 2023	4	Prospective	627	37	> 20% vol	12
Prabhuarj 2019	4	Retrospective	77	3	> 2 mm growth after 24 months	12
Schneider 2016	4	Retrospective	40	3	double initial tumor vol	12 (12–14)
Schumacher 2017	4	Retrospective	30	1	persistent growth	11
Smith 2019	4	Retrospective	177	21	progression	12 (11-16.8)
Soni 2021	4	Retrospective	44	5	≥ 10% vol	12.9 (12–13)
Su 2014	3	Prospective	13	0	> 2 mm in 1 dim^d^ or > 1 mm in 2 dim	12 (11–14)
Tamura 2009	4	Retrospective	74	5	growth	12 (9–13)
Timmer 2011	3	Prospective	100	8	> 2 mm growth in axial plane	11 (9.3–12.5)
Tuleasca 2019	4	Retrospective	5	0	growth	12
VanDeLangenberg 2011	4	Retrospective	33	4	growth	11.6 (10.3–13)
Wangerid 2014	4	Retrospective	128	10	> 2 mm growth	12.3 (11–16)
Wu 2017	4	Retrospective	187	17	> 10% vol	12 (11–13)
Yomo 2012	4	Retrospective	154	12	> 10% vol	12.1 (9–14)

^a^OLE = Oxford Level of Evidence, ^b^treatment failure defined by growth on imaging, ^c^vol = volume increase, ^d^dim = dimensions

### Patient attributes

A total of 5619 patients received GKRS, with primary treatment in 5356 (95.3%) patients and salvage therapy in 263 (4.7%) patients. For those undergoing salvage therapy, the interval between microsurgery and salvage GKRS ranged between 2 and 329 months [20–21]. In these studies, salvage radiosurgery was indicated for regrowth of residual tumors in up to 88% of cases after incomplete resection versus 23% of cases after gross total resection.

For the patients managed with GKRS as their primary treatment, the mean age was 55.7 years (range, 11–91) and 48.3% [95% CI 46.9–49.7%] were males. Using the Koos classification, the proportion of Grade I tumors was 19.2% [1.7–49.2%], II was 19.8% [4.2–42.9%], III was 14.8% [4.7–29.3%], and Grade IV was 49.8% [16.7–83.0%]. In contrast to those treated primarily, patients who received salvage GKRS had a mean age of 52.9 years (range, 15–79) and did not differ from the primary treatment group in the proportions of males (diff. 5.3% [-0.9-11.3%], *p* = 0.09).

When comparing GKRS and microsurgery outcomes, the average monitoring periods varied considerably between studies. For example, one study had a mean post-GKRS follow-up (19.6 ± 5.1 months) comparable to post-surgery monitoring (19.1 ± 4.7 months) (*p* = 0.6) [22], yet another study monitored post-GKRS (43.8 ± 22.8 months) and post-surgery (49.4 ± 29.2 months) for longer durations (*p* = 0.5) [23]. The relative risk of treatment failure in GKRS was 0.4 [0.1–1.3], (*p* = 0.1). As identified by imaging, GKRS treatment failure was 6.1% [4.8–7.7%] in the primary treatment group and 9.0% [5.9–12.8%] in the salvage therapy group (diff. 2.8%, [-0.2-6.9%], *p* = 0.07). Outcomes were further analyzed according to each study’s radiologic threshold for failure as discussed in the methodology. When studies categorized GKRS failure on imaging as an increase in tumor volume over 15%, the failure rate of 3.2% [2.1–4.6%] was significantly less than failure rates seen with the following thresholds: a 10% volumetric increase (diff. 4.8% [2.2–7.8%], *p* = 0.0001), a 20% volumetric increase (diff. 6.0% [3.9–8.1%], *p* < 0.0001), 2 mm tumor growth (diff. 2.6%, [0.1–5.7%], *p* = 0.04), and tumor growth without any size threshold (diff. 3.5% [1.9-5.0%], *p* = 0.0001) (Table 2) The results of GKRS were also reported to be less successful based on the failure criteria of 20% volumetric expansion (9.2% [4.0-16.1%]) versus the non-specific tumor growth (6.7% [4.9–8.8%]) reported frequently in studies (diff. 2.5% [0.5–4.6%], *p* = 0.01).

**Table 2:** Gamma knife radiosurgery failure rates by size

	Tumors (*n*)	Failure Rate (%)	95% CI^a^ (%)
Failure criteria on imaging			
> 2 mm growth	358	5.9	3.7- 8.8
> 10% volume increase	444	8.0	5.6- 10.9
> 15% volume increase	902	3.2	2.1- 4.6
> 20% volume increase	1041	9.2	4.0- 16.1
Non-specific growth	2319	6.7	4.9- 8.8
Tumor volume (cm^3^)			
< 0.8	172	7.3	3.9- 12.2
0.8–1.6	303	5.7	3.4- 8.9
1.6–3.2	933	7.9	3.8- 13.2
> 3.2	119	5.9	1.3- 13.4

^a^CI = Confidence Interval

Treatment failure stratified by tumor volume is shown in Table 2. Compared to the smallest tumor volume, there was no difference in the failure rate for tumors 0.8-< 1.6 cm^3^ (diff. 1.6%, [-2.8-6.8%], *p* = 0.5), tumors 1.6-< 3.2 cm^3^ (diff. 0.6%, [-4.5-4.1%], *p* = 0.8) nor tumors ≥ 3.2 cm^3^ (*p* = 0.6). Tumors 0.8-< 1.6 cm^3^ had similar failure rates to that of tumors 1.6-< 3.2 cm^3^ (diff. 2.2%, [-1.4-5.0%], *p* = 0.2) and tumors ≥ 3.2 cm^3^ (diff. 0.2%, [-4.2-6.3%], *p* = 0.9). Failure rates between tumors of 1.6-< 3.2 cm^3^ and tumors ≥ 3.2 cm^3^ also did not differ (diff. 2.0%, [-4.0-5.5%], *p* = 0.4).

For the patients who experienced GKRS failure during their primary treatment, 74.7% [95% CI 61.6–85.9] required further intervention. Additional GKRS or other SRS techniques were employed in 41.8% [26.9–57.5] of failed cases, while microsurgery was used in 47.2% [36.7–57.9] cases. SRS and microsurgery were employed at comparable frequencies (difference 5.4% [-4.1-14.7%], *p* = 0.3).

In addition to treatment failure, adverse events (AE) associated with GKRS for primary tumor management were reported such as headaches (3.5% [95% CI 0.3–10]) and intratumor cyst formation (6.1% [3.8–9.3]). The most common AE was brainstem edema in 83 patients (8.8% [95% CI 7.1–10.8]) with 25.8% [16.9–36.3] presenting symptomatically. These patients required treatment with steroids (12.5% [1.5–31.7]) or placement of a ventriculoperitoneal (VP) shunt (7.0% [0.8–18.6]). The frequency of using steroids versus VP shunt did not differ (*p* = 0.2).

Trigeminal neuralgia and sensory dysfunction occurred at similar rates in 4.9% [2.8–7.6%] and 4.3% [2.7–6.2%] of patients (*p* = 0.4), respectively. Rates of facial paresis were temporary for 3.2% [1.2–5.9%] and permanent for 2.5% [0.9–4.7%] of cases, while transient facial nerve spasms were at 3.9% [1.5–8.3%]. Hearing preservation rates were evaluated at 1-, 3-, 5-, and 10-years post-treatment with hearing worsening over time (Table 3). Preservation rates declined as much as 57.0% between 1- and 10-year post-treatment evaluations.

**Table 3:** Hearing preservation rates post-Gamma knife radiosurgery treatment

	Hearing preservation rates (%) at:			
Study Year	1 year	3 years	5 years	10 years
Carlson 2013	80.0	55.0	38.0	23.0
Dumot 2022			56.8	45.2
Dumot 2023			69.3	50.9
Johnson 2019	89.8	76.9	68.4	51.40
Klijin 2016		65.0	42.0	
Mousavi 2015	81.0	54.0		
Ogino 2021 (Koos I)		76.6	63.5	27.3
Ogino 2021 (Koos IV)		58.1	50.3	
Paek 2005			46.0	
Pikis 2023			65.0	44.6
Su 2014		77.0	64.0	
Tamura 2009		78.4		

## Discussion

In this study, GKRS failure rates ranged from 3.2 to 9.2%, and 74.7% of treatment failures required additional intervention. The proportion of treatment failure was not associated with primary vs. salvage treatment and did not differ among the smaller volume tumors selected for GKRS. With the various radiologic criteria for tumor growth, most groups had similar failure rates except for those identified with greater than 15% or 20% tumor volumetric expansion. This highlights the difficulty of interpreting outcomes without a uniform threshold for tumor growth. The risk of treatment failure for GKRS was not significantly less compared to that of microsurgery, and treatment failure occurred within 1.6 to 6.2 years on average. The most frequent AE of brainstem edema presented symptomatically and necessitated intervention in 25.8% of cases.

As an established treatment alternative, GKRS demonstrates its efficacy with an overall failure rate of 6.4%. Four studies evaluated surgical resection versus GKRS with the relative risk of treatment failure not differing between the groups. This assessment is congruent with prior literature on smaller VS concluding that microsurgery and GKRS were safe, successful methods for tumor control without clear superiority of one approach [5, 23–25]. GKRS has historically been most effective for growth prevention of small to medium-sized VS < 3.0 cm in diameter or 10.0 cm^3^ in volume, while microsurgery is usually selected for removal of larger tumors [4, 10, 11]. It is recommended that the decision to pursue GKRS or microsurgery be based on treatment goals and factors such as patient’s predictors for tumor growth, surgical risk, and symptom burden [4].

Potential causes of GKRS failure have been identified as tumor volumes > 15 cm^3^ or mass effect with brainstem or ventricular compression [11, 26–29], but there is less consensus on the role of prior resection and radiologic characteristics [10, 29–31]. In Yang et al., the use of GKRS in a post-microsurgical salvage setting was predictive of a higher likelihood of treatment failure [29], yet a study by Yeole et al. concluded that primary vs. salvage status was not significant [10]. In this review of sporadic VS, there was no difference in the failure rate of the primary and salvage groups (*p* = 0.07). This suggests that post-surgical changes do not negatively impact the success of GKRS, which is valuable knowledge when considering salvage treatment. However, this study failed to corroborate an association between higher failure rates and larger tumor volumes likely due to the sampling bias of smaller VS typically being selected for GKRS.

In the scenarios when GKRS fails, additional treatment is needed to address tumor progression. Retreatment with SRS or surgical resection occurred at similar rates to achieve tumor control, which was expected given the lack of consensus on how to retreat VS after failed radiosurgery [32]. Although an increased risk of facial and trigeminal dysfunction has been reported after repeat GKRS, there is still preserved cranial nerve function in most patients, and it is suggested that repeated GKRS can be advantageous due to the cumulative radiation effects on tumors [32–33]. Gamma knife retreatment has effective tumor control with low morbidity and is recommended for small-medium sized VS (< 3.0 cm in diameter) [32, 34, 35]. Selection criteria for retreatment is typically guided by size with larger tumors surgically resected, but cranial nerve dysfunction, adverse radiation effects and tumor adhesions from primary treatment may be limiting factors [32, 34].

Regarding AE associated with GKRS, it was not surprising that brainstem edema was the most frequently observed due to the proximity of the brainstem to the vestibulocochlear nerve. The brainstem has a suggested single dose tolerance of 12–15 Gy (Gy), and this threshold is usually easily met as the prescription dose for vestibular schwannoma typically ranges from 10 to 13 Gy [36–38]. The radiation-induced changes on imaging can include peritumoral edema, hemorrhage, pseudoprogression, or true tumor progression that may warrant further treatment as seen in 25.8% of the patients in the current study [38–39]. Patients who presented with edema-associated symptoms were commonly managed with a short steroid course or a VP shunt, which is why patients should be monitored for this complication appropriately [40–42].

Among the included studies, the rate of new-onset trigeminal neuropathy was variable given the wide range of tumor size and radiation delivered [43, 44]. The incidence of neuralgia (4.9%) and sensory deficits (4.3%) aligns with prior literature (0.0–14.0%) and demonstrates the ability of GKRS to preserve cranial nerve function [9, 45–47]. Furthermore, facial nerve function was intact in over 95% of cases which is superior to the preservation rate reported after excision (68.0%) and can greatly improve quality of life [48–49]. However, it is difficult to avoid hearing loss with radiation exposure to the cochlea as evidenced by declining serviceable hearing over time (Table 3) [44]. Established risk factors such as increasing tumor size, patient age, cochlea dose, and GR grade can greatly influence hearing outcomes [50–53]. Hearing preservation should be monitored long-term since most patients experience a decline by 10 years post-treatment, but a recent review found that GKRS has greater efficacy in stable hearing capability compared to fractionated radiotherapy and surgery [54].

With considerable study heterogeneity, several limitations were encountered. The search criteria limited the patient population and excluded those with concurrent intracranial tumors to minimize confounding treatments. Many studies failed to subcategorize patients by treatment phase e.g., primary vs. salvage, and therefore were removed from the analysis. We aimed to assess the conformity index and any potential relationship with the frequency of toxicities but were unable to due to sparse reporting of the conformity index. Regression analysis also could not be performed to evaluate the relationship between risk factors (e.g. tumor size) and treatment failure due to insufficient data. There was inconsistent reporting of pre-existing and post-treatment auditory and vestibular side effects. Only post-treatment data on adverse events was collected in this study.

## Conclusions

This study highlighted that GKRS treatment failure was not impacted by the primary versus salvage timing and there were similar outcomes among the smaller tumors evaluated. There was variability in the failure rates when categorized by the common tumor growth thresholds which prompts consideration of a uniform threshold for post-treatment growth. GKRS is an excellent treatment option for sporadic VS, with outcomes minimizing cranial nerve dysfunction, radiation toxicity, and need for retreatment.

## Electronic supplementary material

Below is the link to the electronic supplementary material.

Supplementary Material 1

## Data Availability

Data can be accessed upon reasonable request to the corresponding.
